# Addressing Women's Health Care Needs During Pediatric Care

**DOI:** 10.1089/whr.2021.0016

**Published:** 2021-07-09

**Authors:** Rachel N. Caskey, Sarah E. Olender, Alejandra Zocchi, Cara J. Bergo, Keriann H. Uesugi, Sadia Haider, Arden S. Handler

**Affiliations:** ^1^ Department of Medicine, University of Illinois at Chicago, Chicago, Illinois, USA.; ^2^ Center of Excellence in Maternal and Child Health, School of Public Health, University of Illinois at Chicago, Chicago, Illinois, USA.; ^3^ Department of Obstetrics and Gynecology, University of Chicago, Chicago, Illinois, USA.

**Keywords:** postpartum care, contraception, pediatric care, well-baby care

## Abstract

***Objective:*** To determine if the use of a simple self-administered *Postpartum Questionnaire for Mothers (PQM)* at the well-baby visit (WBV) increased the proportion of women who received health care and contraception by 6 months postpartum (PP).

***Methods:*** This was a single-site, system-level, intervention. Women were recruited from the pediatric clinic when presenting with their infants for a 2-month WBV. During phase 1 of the study, a control group was enrolled, followed by an 8-week washout period; then enrollment of the intervention group (phase 2). During phase 2, the *PQM* was administered and reviewed by the pediatrician during the infant's visit; the tool prompted the pediatrician to make a referral for the mother's primary or contraceptive care as needed. Data were collected at baseline and at 6 months PP, and additional data were extracted from the electronic medical record.

***Results:*** We found that PP women exposed to the *PQM* during their infant's WBV were more likely to have had a health care visit for themselves between 2 and 6 months PP, compared with the control group (relative risk [RR] 1.66, [confidence interval (CI) 0.91–3.03]). In addition, at 6 months PP, women in the intervention group were more likely to identify a primary care provider (RR 1.84, [CI 0.98–3.46]), and more likely to report use of long-acting reversible contraception (LARC) (RR 1.24, [CI 0.99–1.58]), compared with women in the control group.

***Conclusion:*** A simple self-administered *PQM* resulted in an increase in women's receipt of health care and use of LARC by 6 months PP.

***Clinical Trial Registration:*** Use of a reproductive life planning tool at the pediatric well-baby visit with postpartum women, NCT03448289.

## Background

The period after birth of an infant is a critical time for new mothers, infants, and families.^1^ The postpartum (PP) period, sometimes referred to as the fourth trimester, is a time of transition for both mothers and infants, and is accompanied by higher than average risk for morbidity or mortality.^2,3^ Many women experience morbidity during pregnancy (*e.g.*, hypertension, diabetes), which often continues into the PP period, making the receipt of health care in the year after delivery particularly important.^4^ Complications from chronic disease, rather than obstetric complications, are now the leading cause of maternal morbidity in the United States.^5^

In addition, women who have had a recent pregnancy are at increased risk of an unintended pregnancy, compared with other women of reproductive age,^6^ with rates up to 44% in the first PP year.^7^ Pregnancies with a short interpregnancy interval (within 18 months of delivery) have been associated with an increased risk of preeclampsia, preterm birth, and low birth weight.^8,9^ While some women have access to contraception immediately after delivery, provision of contraception varies and many women do not receive timely PP contraception.^10^ Improved access to PP care and contraception is needed to improve maternal outcomes and help women achieve desired birth spacing.^11,12^

Although recommendations have recently changed, traditionally, PP care has taken place at ∼4–6 weeks after delivery. However, attendance at the 4–6-week PP visit is low, with studies showing 11%–46% of women not attending a PP visit.^13–18^ In contrast, well-baby visits (WBVs) begin shortly after delivery and are highly utilized. In 2011–2012, approximately 90% of U.S. infants received visits during the first year of life.^19^ Because many women are more likely to obtain care for their infants, compared with their own care, they are likely to attend multiple visits in the pediatric setting during the PP period. As such, the WBV is increasingly acknowledged as a site of care where maternal health issues can be identified and addressed.^20–22^

The objective of this study was to test the impact of an innovative system-level intervention in which PP women completed a brief self-administered *Postpartum Questionnaire for Mothers (PQM)* during their infants' 2-month pediatric visit, on subsequent receipt of primary health care and contraception by 6 months PP, compared with usual care.

## Methods

### Study design and sample

**FIG. 1.**
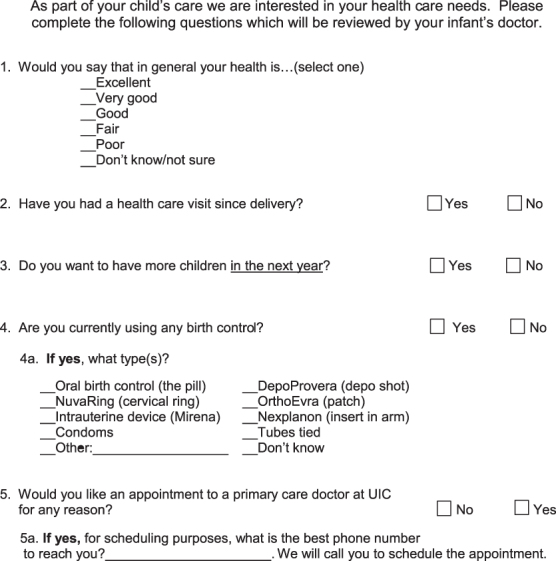
Postpartum care questionnaire for mothers.

This was a single-site, system-level, pilot study. The *PQM* is a one-page, five-question self-administered tool that included questions on general health, use of contraception, and desire for health care (Fig. 1). The *PQM* was designed to be briefly reviewed by the pediatrician during an infant's visit, and prompt the pediatrician to make a referral for the mother's care, when needed.

This study had two phases. Phase 1 consisted of control group enrollment, followed by an 8-week washout period; this was followed by enrollment of the intervention group (phase 2). During both phases, women were recruited from the general pediatric clinic when presenting with their infants for a 2-month WBV. During phase 1 (control) women were asked to enroll in a control group for a general study about women's health, but were not explicitly informed of the purpose of the study, to avoid influencing health behaviors.

During phase 2 (intervention), the self-administered *PQM* was implemented in the general pediatric clinic. For convenience, the *PQM* was paired with the self-administered Edinburgh Postpartum Depression Screen (EPDS) as the EPDS is routinely administered to all PP mothers in the clinic. Results of the EPDS were not recorded for this study. Both tools were provided upon check-in to all mothers of infants scheduled for a 2-month WBV, and mothers were instructed to complete the tools while waiting for the visit.

During the WBV, the pediatrician briefly reviewed the *PQM* and offered the mother a referral for primary care if desired. Primary care options included internal medicine, family medicine, or obstetrics/gynecology, based on the woman's preference. For convenience, women who desired a visit for themselves were offered the opportunity to either coschedule their visit on the same day as the infant's next WBV, or request the first available appointment, based on preference.

Pediatricians received formal training on the use of the *PQM* before the start of the intervention through a 1-hour didactic training session. Research staff communicated with pediatricians and clinic staff weekly during the first 4 weeks of the study, and monthly thereafter, to answer questions and encourage consistent provider use of the tool. During the training, pediatricians were instructed on how to initiate a conversation with women using the tool and how to initiate the referral process. Pediatricians were not expected to provide care or contraceptive counseling to mothers, only to engage women in a brief discussion about their need for health care and offer a referral to primary care as desired. The pediatricians were trained to complete the *PQM* by indicating in a checkbox whether a woman desired a referral for care.

### Data collection

Although all women in the pediatric clinic at the 2-month WBV were given the *PQM* to complete, only women who consented to be in the “study” were enrolled. Study staff approached PP women waiting for the 2-month WBV, screened for eligibility, and administered informed consent before the WBV. Women in the intervention group were recruited after they completed the *PQM* in the waiting room.

Survey data were collected at baseline (2 months PP) and 6 months PP for both the control and intervention groups. Baseline data collection surveys were self-administered in the clinic at the time of enrollment; the 6-month surveys were administered by phone. The surveys were identical for both groups except during the 6-month follow-up survey the intervention group received questions about their experience completing the *PQM* and their interactions with the pediatrician about their own health care needs. Medical records were reviewed at 6 months PP to determine receipt of health care services and contraception since delivery.

This was a system-level intervention, thus, all women presenting for the 2-month WBV during phase 2 were exposed to the *PQM* and offered a referral if needed or desired, irrespective of their consent to participate in the study. *PQM* data were collected for all women; individual survey data were only collected for women who consented to participate in this study. This study was approved by the University of Illinois at Chicago Institutional Review Board (IRB).

### Sample and sample size

Eligible participants included PP women who were between the ages of 15 and 49 years, spoke either English or Spanish, and received their own health care at the University of Illinois Health and Hospital Systems (UIH). Women who were currently pregnant were excluded. Target enrollment for this pilot study was 50 women in each of the two arms (control and intervention) based on how many participants could be enrolled during the study period. The achieved sample size of 100 was estimated to provide a stable estimate of the intervention's effect on primary care receipt by 6 months PP.

### Outcome measures

The primary outcome, receipt of primary care services for women between 2 and 6 months PP, was ascertained both by self-report during the 6-month phone interview and by medical record review, to minimize missing data. Evidence of a visit from either source was counted as receipt. In the event that a follow-up survey was not obtained and there was no evidence of visit in the medical record, the participant was counted as no follow-up visit.

Study eligibility criteria included that women receive their own care at the UIH medical center; thus, our assumption is that most care received during the follow-up period would be available in the medical record. We conducted a sensitivity analysis to assess if this assumption made an impact on the study results. The secondary outcomes, utilization, and type of contraception by 6 months PP, were assessed similarly.

### Statistical analysis

Baseline characteristics were assessed for equivalency across groups using chi-square tests for categorical and *t*-tests for continuous variables. An intent-to-treat analysis was performed. Relative risks and risk differences were calculated with 95% confidence intervals (CIs) to estimate the effect of the intervention on primary and secondary outcomes using a generalized linear model that adjusted for baseline characteristics not equivalent between groups. Data were analyzed in SAS 9.4 (Cary, NC).

## Results

One hundred women were enrolled in the study (50 control and 50 intervention); an additional 57 women completed the *PQM* but did not enroll in the study. Phase 1 enrollment occurred between January and April of 2018, followed by an 8-week wash-out period; phase 2 enrollment occurred between July and November of 2018. The women in both groups were similar; the majority were between 19 and 34 years (74% intervention, 75.5% control) and not married (66% intervention, 70% control) but living with a male partner (74% intervention, 60% control) (Table 1). Most women were African American (46% intervention, 62% control) or Latina (32% intervention and 24% control) and had health insurance either through Medicaid (60% intervention, 52% control) or private insurance (40% intervention, 46% control) (Table 1).

**Table 1.** Baseline Characteristics of Intervention and Control Groups

	Intervention group (*n* = 50), *n* (%)	Control group (*n* = 50), *n* (%)	*p*
Maternal age (years)			
<19	1 (2)	3 (6)	0.31
19–34	37 (74)	37 (76)	
35+	12 (24)	9 (18)	
Marital status			
Married	17 (34)	15 (30)	
Not married	33 (66)	35 (70)	0.67
Living situation			
Living with male partner	37 (74)	30 (60)	
No living with male partner	13 (26)	20 (40)	0.14
Education			
High school or less	16 (32)	19 (38)	0.53
More than high school	34 (68)	31 (62)	
Work status			
Working	20 (40)	22 (44)	0.80
Not working	15 (30)	12 (24)	
On maternity leave	15 (30)	16 (32)	
Race/Ethnicity			
African American	23 (46)	31 (62)	0.35
Latina	16 (32)	12 (24)	
White	7 (14)	3 (6)	
Asian	4 (8)	3 (6)	
Other	0	1 (2)	
Health insurance during pregnancy			
Medicaid	30 (60)	26 (52)	0.48
Private	20 (40)	23 (46)	
Other	0	1 (2)	
Health insurance currently			
Medicaid	30 (60)	26 (52)	0.48
Private	20 (40)	23 (46)	
Other	0	1 (2)	
Has a primary care provider			
Yes	39 (78)	38 (78)	0.60
No	11 (22)	11 (23)	

The majority of women had resumed sexual intercourse before 2 months PP (62% intervention, 54% control); however, only half were using *most effective (long-acting reversible contraception, LARC)* or *moderately effective (pills, patch, ring, or injection)* contraception (56% intervention, 58% control), and nearly all did not intend to have a pregnancy for at least a year or more (Table 2). Over 30% of women in each group reported that this recent pregnancy was either mistimed or not desired (Table 2). Most were offered some form of contraception after delivery, although just over half (53.9% intervention group and 48.7% control group) reported using a contraceptive method after delivery. There were no statistically significant differences in baseline data related to pregnancy history or contraception use.

**Table 2.** Baseline Pregnancy History, Postpartum Visit Attendance, and Contraception Use

	Intervention group (*n* = 50), *n* (%)	Control group (*n* = 50), *n* (%)	*p*
Number of pregnancies (including most recent)			
1	13 (26)	14 (28)	0.48
2–3	24 (48)	27 (54)	
4+	13 (26)	9 (18)	
Number of children living with mother, mean (SD)	1.8 (1)	1.9 (1)	0.49
Recent pregnancy intention			
Intended	31 (66)	28 (65)	0.67
Mistimed	4 (9)	6 (14)	
Not wanted	12 (26)	9 (21)	
Gestation age at delivery			
Preterm (<37 weeks)	6 (12)	13 (27)	0.07
Term	44 (88)	36 (74)	
Future pregnancy desire			
No more pregnancies	21 (42)	24 (48)	0.55
Pregnant in the next 6 months	1 (2)	0	
Pregnant in the next 1–2 years	8 (16)	8 (16)	
Pregnant in 2 or more years	17 (34)	12 (24)	
Do not know	3 (6)	6 (12)	
Resumed sexual intercourse			
Yes	31 (62)	27 (54)	0.42
No	19 (38)	23 (46)	
Weeks after delivery that sexual intercourse resume, mean (SD)	6.2 (2)	5.7 (2)	0.33
Offered contraception at delivery			
Yes	39 (78)	39 (78)	0.60
No	11 (22)	10 (20)	
Do not know	0	1 (2)	
Selected and started a form of contraception after delivery			
Yes	21 (54)	19 (49)	0.65
No	18 (46)	20 (51)	
Currently using contraception, by effectiveness			
Most effective (intrauterine device, implant)	11 (22)	17 (34)	0.53
Moderately effective (pills, patch, ring, or injection)	17 (34)	12 (24)	
Least effective (condoms)	8 (16)	7 (14)	
No contraception	14 (28)	14 (28)	
Attended PP visit			
Yes	45 (90)	42 (84)	0.38
No	5 (10)	8 (16)	

PP, postpartum; SD, standard deviation.

All women in the intervention group completed the *PQM* (*n* = 50). Based on the pediatrician's indication on the *PQM,* 11 women (22%) expressed an interest in a referral for care; all 11 women were contacted, 4 were scheduled for a health care visit, and the remaining 7 women opted not to schedule an appointment. Similarly, among *nonparticipants* who were exposed to the system-level intervention and completed the *PQM* but did not enroll in the study, 12 women (21%) expressed interest in a referral, all 12 were contacted, and 4 opted to schedule an appointment.

At the 6-month follow-up, 78% of the intervention women recalled filling out the *PQM* and of these, 58% recalled having a conversation with the pediatrician about their own need for care (Table 3).

**Table 3.** Referral for Care for Postpartum Women Exposed to Postpartum Questionnaire for Mothers

	Intervention group (*n* = 50), *n* (%)	Nonparticipant group^a^ (*n* = 57), *n* (%)	Total (*n* = 107), *n* (%)
PQM completed	50 (100)	55 (96)	105 (98)
Reported need for care			
Patient interest in referral for a primary care visit	11 (22)	12 (21)	23 (21)
Contacted for an appointment	11 (22)	12 (21)	23 (21)
Primary care visits scheduled	4 (8)	4 (7)	8 (7)
Results from 6-month follow-up			
Woman recalls completing the PQM	35 (78)	N/A	N/A
Recalled pediatrician discussing need for a referral for care	25 (56)	N/A	N/A

^a^ Did not enroll in study; completed PQM as part of system-level intervention.

N/A, not applicable; PQM, Postpartum Questionnaire for Mothers.

Women in the intervention group were more likely to have received a health care visit between 2 and 6 months PP, compared with the control group (relative risk [RR] 1.66, 95% CI [0.91–3.03]) (Table 4). In addition, women in the intervention group were more likely to identify a primary care provider by 6 months PP, compared with women in the control group (RR 1.84, 95% CI [1.00–3.46]). Although use of any contraception was similar between the intervention and control groups, twice the number of women in the intervention group reported use of LARC (32% vs. 16%, respectively). The lower limits of the 95% CIs for the crude and adjusted relative risks and risk differences of all the outcomes, with the exception of any contraception by 6 months, was slightly below the null indicating that each measure of association was trending toward statistical significance. A sensitivity analysis where the primary and secondary outcomes were defined as missing if there was no medical record evidence of a health care visit or receipt of contraception showed similar results.

**Table 4.** Health Care Visit and Receipt of Contraception at 6 Months Postpartum

	Intervention (*n* = 50)	Control (*n* = 50)	Adjusted^a^ RR (95% CI)	Adjusted^a^ RD (95% CI)
A health care visit between 2 and 6 months PP	38 (76%)	29 (58%)	1.66 (0.91–3.03)	0.16 (−0.02 to 0.35)
Identifies primary care provider^b,c^	33 (73%)	22 (55%)	1.84^c^ (1.00–3.46)	0.19 (−0.01 to 0.39)
Use of any contraception	37 (74%)	36 (72%)	0.78 (0.32–1.88)	−0.04 (−0.21 to 0.12)
Use of LARC	16 (32%)	8 (16%)	1.24 (0.99–1.58)	0.16 (−0.004 to 0.32)

^a^ Adjusted for weeks PP, which was nonequivalent across groups.

^b^ Fifteen participants missing.

^c^ Adjusted for weeks PP at follow-up.

CI, confidence interval; LARC, long-acting reversible contraception; RD, risk difference; RR, relative risk.

## Discussion

In this system-level pilot project, we found that PP women exposed to a brief self-administered *PQM* during their infant's WBV, were 66% more likely to have had a health care visit between 2 and 6 months PP, compared with women who were not exposed to the *PQM*. In addition, women in the intervention group were 84% more likely to identify a primary care provider, and 24% more likely to report use of LARC, by 6-month PP, compared with women in the control group.

The effect sizes of the adjusted relative risks and risk differences found in this study are meaningful in a clinical setting and trended toward significance. Indeed, for every 100 women seen in the intervention period, there were 16 more women who had a health care visit between 2 and 6 months, 19 more women who identified having a primary care provider, and 16 more women who reported use of LARC by 6 months compared with women seen during the control period. It is likely that the inclusion of the null in the 95% CIs is a result of the small sample size in this pilot study. A larger study should seek to replicate these effect sizes with adequate power and sample size.

Many PP women do not receive timely care and contraception during the PP period, putting them at risk for untreated health problems or unintended pregnancy.^23^ Pediatric health care visits are a site where maternal health issues can be identified, and in some cases, directly addressed. The American Academy of Pediatrics (AAP) Bright Futures guidelines for health supervision encourage providers to assess maternal well-being as part of routine care of infants.^24^ Nationally, pediatricians now routinely act as a screening agent and source of referral for PP depression among new mothers.^25^ In over half of states, including Illinois, the state Medicaid agency reimburses pediatricians for screening new mothers for depression.^26^

Ideally, reviewing women's health and contraceptive needs could occur at every WBV during the first year of life; however, we selected the 2-month WBV as the focal point of our intervention because this visit should typically occur after the traditional 4–6 week PP visit. As this was a proof-of-concept study, we did not want to interfere with any scheduled PP care. Instead, we focused on women's need for care beyond 8 weeks PP to ensure all women were given the opportunity for care, regardless of whether they had obtained a PP visit.

The delivery of newborn care is an important touch point for addressing women's health care needs as well. Our study finds that an intervention in which a mother completes a brief questionnaire about her health needs and the pediatrician affirms these needs, positively impacts women's receipt of care during the extended PP period. This is critical as risks to women do not end at 4–6 weeks PP. For example, in Illinois, of the pregnancy-related deaths in 2015, 14% of deaths occurred during pregnancy, 53% occurred within 42 days PP, and 33% occurred between 43 and 364 days of the most recent pregnancy.^6,27^

In this study, 87 of the women (intervention and control) attended their PP visit, which is substantially higher than what has been found with other mostly low-income populations.^13–18^ While this high PP attendance rate might suggest that women would not desire another visit within 6 months PP, 22% of women expressed a desire for, or need for, care with a primary care provider between 2 and 6 months PP. This study suggests that the act of a pediatrician asking about women's health needs may reinforce the importance of care in the extended period after delivery and may consciously, or subconsciously, link the importance of maternal health with infant health.

While a model for providing care to both mother and infant together may exist for women receiving care from a family physician,^28^ for most families, adult care is separate from pediatric care. Family physicians provide 15%–17% of primary care visits for children under the age of 4 years, whereas pediatricians provide the remainder.^29,30^ Our study is the first to document the positive effect of the use of a questionnaire focused on women's general health and contraceptive needs, to generate a referral for maternal care, among mothers attending a pediatric WBV.

Our study had limitations. Because this was intended to be a system-level intervention, we were unable to randomly assign women to receive the intervention. We recruited women into each group in close succession, with an adequate wash-out period (8 weeks) between groups to ensure a woman would not be recruited into both groups. We did not collect data on the pediatricians, and so we are unable to determine if physician attributes or conversational approaches contributed to women seeking care for themselves. However, pediatricians consistently used the tool during the study period suggesting the tool was relatively easy to use and did not interfere in care.

This was a pilot study with a small sample size intended to provide estimates of effect size for calculating the sample size for a larger trial. We minimized missing data for the primary and secondary outcomes by including medical records, and a sensitivity analysis showed similar results if lack of evidence of the outcomes in the medical record were counted as missing.

## Conclusion

Our findings suggest that many mothers desire to be connected to care in the PP period and that WBV are an opportunity to identify such mothers and link them to care. The health and well-being of newborn infants is intrinsically tied to the health and well-being of mothers. Up until now, the approach to women's health care at the infant's WBV has focused on discrete issues such as PP depression. Moving forward, pediatric visits may be an important opportunity to identify and address mothers' care needs in the early and extended PP periods.

## Abbreviations Used

AAP: American Academy of Pediatrics
CI: confidence interval
EPDS: Edinburgh Postpartum Depression Screen
IRB: Institutional Review Board
LARC: long-acting reversible contraception
N/A: not applicable
PP: postpartum
PQM: postpartum questionnaire for mothers
RD: risk difference
RR: relative risk
SD: standard deviation
UIH: University of Illinois Hospital
WBV: well-baby visit
